# Supplementary material for: Recommendations for the Development of Family-Based Interventions Aiming to Prevent Unhealthy Changes in Energy Balance-Related Behavior during the Transition to Parenthood: A Focus Group Study
Source: Nutrients. 2022 Jun 4;14(11):2346. doi: 10.3390/nu14112346 (PMC9182952; doi:10.3390/nu14112346)
Supplement: Supplementary file 1 [file nutrients-14-02346-s001.zip › nutrients-1695081-supplementary.pdf]

Supplementary Materials

Table S1. Checklist for the development of intervention strategies aiming to maintain or obtain healthy eating behavior and physical activity in expecting and first-time parents.

INTERVENTIONS DURING PREGNANCY AND POSTPARTUM AT AN individual level		
	Item number	Recommendation
**WHAT**		
Content – general	1	*Pregnancy and postpartum:*<br>Ensure advices are specific, filtered and not too much/overwhelming.<br>Included messages about what is still possible during pregnancy and give replacement messages.<br>Include practical tips and tricks.<br>*Postpartum:*<br>Bundle information together with information for the child.<br>Add time-efficient/time-saving information/message.
Content – nutrition and physical activity	2	*Nutrition:*<br>Nutrition guidelines.<br>Menu and recipe inspiration.<br>Product information.<br>*Physical activity:*<br>Messages encouraging physical activity (e.g., walking) instead of sport.<br>Adjusted exercises and activities for pregnant women/couples/with child.<br>Exercises to do at home.<br>Encourage active transportation.
Content - Adaptations	3	Adapt information per trimester during pregnancy or period postpartum (early vs. later postpartum).<br>Adapt information to sex.
Materials	4	Materials have to be attractive, visual and not outdated.
**Intervention aims**		*Examples of intervention aims can be found in Appendix 2.*
Socio-ecological levels: individual behavior change	5	Focus on improving/shaping knowledge and following self-regulation skills:<br>- Planning and anticipation<br>- Self-belief<br>- Self-control and self-management<br>- Self-care<br>- Self-discipline<br>- Self-efficacy
Behavior change techniques	6	Make use of following behavior change techniques:<br>- Give information about health advantages and consequences<br>- Add associations (prompts and cues)<br>- Include feedback and monitoring (on outcome and behavior)<br>- Support goal setting (outcome and behavior) and action planning

		- Help with (re)framing of behavior<br>- Focus on habit formation/retaining habits (men)<br>- Add rewards (social and/or material incentives)<br>- Include identification as role-models
Changes at interpersonal level	7	How may the social environment be included?<br>- Partner support: include how partners can motivate each other.<br>- Professional support: make sure healthcare providers who deliver the intervention give consistent advice, monitoring and support. Include emotional support (e.g., active listening, being available). Adjust professional support to the need of young parents (e.g., online or at home visits).<br>- Social environment: Include the broader social environment of the couple to provide practical support. Include messages/focus on changing social norms, knowledge and misperceptions about nutrition, physical activity and sedentary behavior during pregnancy and postpartum.
Changing environmental level	8	Include a focus on a healthy home food environment.
**Intervention characteristics**		
Accessible	9	Make sure the intervention is easy accessible. People need to know where to find the right information with low efforts. Strive for a realistic and not too time intensive intervention which is time and place independent.
Approach	10	Deliver and communicate about the intervention in a non-preachy or non-forced way, without obligations, not too pushy and without giving guilty feelings when people do not succeed in certain parts of the intervention.
Communication	11	Make sure the advice is scientific correct and clear, consistent, and not too much/overwhelming. Use a humorous and funny communication style (to men).
Reliable	12	Add source of the information.
Personalized	13	Provide the opportunity to customize the amount and timing of information. Make sure people can go through the intervention at their own tempo and linked with their own needs.
**HOW**		Mode of delivery and reachability
Individual	14	- Decide how the population will be supported at an individual level (indirect and/or face-to-face)
Group	15	- Decide if a group level to deliver intervention will be included (e.g., workshops)
Population level	16	- Decide how the intervention will be delivered on a population-level (broadcast media, digital media, print media,…)
	17	Centralize the intervention in one platform and use a combination of both an indirect and face-to-face approach to reach the population of (expecting) parents and deliver the intervention.
**WHERE**		
Setting	18	In which setting is the intervention implemented:

		- Home setting: include advice/activities easy accessible at home.<br>- Sport club setting: adjusted courses for pregnant women or couples.<br>- Work setting interventions: adjusted work for pregnant women, flexibility (place, time) during working days.<br>- Restaurants and supermarket setting: focus on product information.<br>- Existing care: incorporate interventions in existing care pathways.
**WHEN AND HOW MUCH**		
When	19	Start of intervention:<br>- Pre-pregnancy: healthy habits in general.<br>- Beginning of pregnancy: focus on nutrition.<br>- Middle of pregnancy: focus on physical activity.<br>- End of pregnancy: focus on physical activity and early postpartum.
How much	20	Decide on the timing of delivery of the information/intervention (during pregnancy/postpartum).<br>Include regularity and follow-up.<br>Decide if a possibility for participants to look for information when needed or if a predefined timing of the intervention will be used.<br>Adapted the information to the period during pregnancy/postpartum and to the couple's needs.
**FOR AND FROM WHOM**		
Participants	21	*Pregnancy:*<br>Couple-based interventions: include both women and men, but with a different intervention aim.<br>*Postpartum:*<br>Family-based interventions. Include the child in the intervention.
Intervention providers	22	Decide if healthcare professionals will be included in the intervention for personal support?<br>Decide which healthcare professionals will be included. Look for professionals who are educated about nutrition and physical activities and who have knowledge about the barriers related with parenthood, otherwise train them in these topics.<br>Choose for one centralized person to communicate about the intervention and who can refer to specialists if needed.
Partner support	23	Include pregnant partner in order to reach men.
Peer support	24	Decide if and how community building will be included in the intervention (e.g., social support of friends and family, use of role models, contact with other pregnant women/expecting fathers).

Table S2. Intervention aims for changes at an individual, interpersonal, environmental and policy level.

	Suggested changes	During pregnancy	Postpartum
**Individual behavior change**			
Shaping knowledge	Information about health consequences	*e.g., explain why it is important, explain health benefits for the baby*	*e.g., explain health benefits of moving (mental benefits, more energy,...)*
	Feedback on behavior	*e.g., taking into account actual behavior and evaluate what can be changed*	*e.g., goal setting, reminders and (personal) follow-up*
	Feedback on outcome of behavior	*e.g., monitoring of body weight*	
Shaping self-regulation skills	Associations (prompts and cues)	*e.g., reminders to be physically active*	*e.g., focus on food child, subscription for animal park*
	(Self-)monitoring of behavior	*e.g., input physical activities*	*e.g., monitoring of kcal you burn while walking, confrontation with changes in behavior*
	Self-monitoring of outcome of behavior	*e.g., weighing at home*	
	(Action) planning	*e.g., meal planning, preparing for what is coming, time management*	*e.g., fixed appointments (with physiotherapist), meal planning/anticipation.*
	Goal setting (behavior)	*e.g., walking goals*	*e.g., easy and specific; "... moving/day"; "... drinking/day"*
	Goal setting (outcome)	*e.g., "glucose screening test", "being fit for delivery and child", "mental benefits"*	*e.g., body weight*
	Habit formation	*e.g., creating certain habits before delivery*	*e.g., make healthy behavior new habit*
	Retaining habit	*e.g., support men not to change their habits*	
	Re-attribution	*e.g., "sometimes things are out of your control"*	*e.g., "things will get better when...", "If I had more time..."*
	Identification of self as role model		*e.g., child health as a motivator*
**Changing social support and social networks**			
Changing professional support	Advice and support	*e.g., consistent advice between healthcare providers, giving the advice and support, monitoring of behavior/outcomes*	*e.g., follow-up by midwife, dietician,… if needed*
	Opportunities		
	Adjusted to the needs of young parents		*e.g., home-visits, child friendly physiotherapist*
	Social incentives	*e.g., positive enforcement*	
Changing social norms	Knowledge of social environment	*e.g., no comments/info from others; knowledge of people*	

		from catering services, restaurants, bakery,...	
Changing and introducing partner support	Focus on how couple can motivate each other	*e.g., support, solidarity of partner*	
Support and knowledge of a wider social environment	Social support (practical)	*e.g., someone to help with groceries, cooking*	*e.g., practical help from friends/family (bringing food). Social network to count on.*
	Social support (emotional)	*e.g., listening, being available*	
	Support from peers	*e.g., advice from other pregnant women*	
	Social comparison		*e.g., group challenges*
Support for child, maternal and household care	Child care		*e.g., network for child care (if no support from parents is possible)*
	Practical care at home		*e.g., cooking and cleaning help. Also after maternity leave.*
**Changing environmental conditions**			
Home food environment	Adding objects to/restructure the physical environment	*e.g., nudging, food boxes*	*e.g., encourage healthy home food environment*
Broader physical environment	Adjusted facilities	*e.g., facilities to rest/do a nap*	*e.g., child friendly sport clubs and restaurants*
	Mobility-friendly environment	*e.g., safer/presence of cycle lanes*	
**Policy and organizational changes**			
Policy changes	Changes in existing care		*e.g., multidisciplinarity, elaborating work of midwife, nutrition and physical activity incorporating in standard care*
	Guidelines from authority		*e.g., "forced me-time"*
	Parental leave		*e.g., increase parental leave (or option to choose who takes parental leave)*
Economical changes	Discounts	*e.g., cheaper pregnancy yoga sessions, discount on certain foods*	
	Reimbursements	*e.g.,reimbursed coachings*	*e.g., personal coaching sessions instead of maternity fee, reimbursed counseling sessions*
Changes on an organizational level	Child care		*e.g., increase opening hours day care*
Work related adjustments	Working hours and workload	*e.g., not full-time, shorter working days, flexibility*	*e.g., more flexibility at work, working less/not full time*
	Work place	*e.g., more home work*	
